# Opportunistic muscle density assay during CT lung cancer screening for low muscle quality evaluation in older adults: a multicenter study

**DOI:** 10.1007/s40520-025-02933-9

**Published:** 2025-02-22

**Authors:** Xin Chen, Xifa Gao, Rongzhou Wang, Zicheng Wei, Jiangchuan Wang, Miaomiao Wang, Chao Xie, Xiao Chen

**Affiliations:** 1 Department of Radiology, Longhua Hospital Shanghai University of Traditional Chinese Medicine, 725 wanping south road, Shanghai, 200032 China; 2 Department of Radiology, The Affiliated Hospital of Nanjing University of Chinese Medicine, Nanjing, 210029 China; 3 Department of Radiology, The Second Affiliated Hospital of Soochow University, Suzhou, 215004 China; 4 Center for Musculoskeletal Research, School of Medicine and Dentistry, University of Rochester, Rochester, NY 14642 USA

**Keywords:** Muscle attenuation, Computed tomography, Lung cancer screening, Muscle‒spleen ratio

## Abstract

**Background:**

Intramuscular adiposity, which can be reflected by muscle computed tomography (CT) attenuation, may be a marker of sarcopenia. This study aimed to investigate muscle attenuation across the life course and thresholds of muscle attenuation for evaluating low muscle quality in older adults.

**Methods:**

This retrospective multicenter study included 9701 subjects aged 20 years and older who underwent CT lung cancer screening from 2019 to 2021 at our institutions in cohort 1. Muscle attenuation (Hounsfield units [HUs]) of the bilateral erector spinae and spleen attenuation at the middle level of the T11 vertebra were measured. The T score, which is analogous to that used to define osteoporosis, was calculated on the basis of absolute muscle attenuation and the muscle‒spleen ratio (M/S). A T score < -2.5 was used to define low muscle density. The cutoff points for muscle CT attenuation and M/S were subsequently calculated to define low muscle density. Another cohort (cohort 2) of 2006 subjects aged 50 years or older was included to explore the association between low muscle quality and vertebral compression fracture (VCF).

**Results:**

The mean [SD] age of cohort 1 was 51.8 [15.5] years, and 5896 [60.8%] men were included. The mean [SD] age of cohort 2 was 62.4 [9.6] years, and 1162 [57.9%] men were included. Multiple linear regression analysis revealed that age was associated with muscle CT attenuation (β = -0.19, 95% confidence interval (CI): -0.21 to -0.18) and the M/S ratio (β = -0.004, 95% CI: -0.004 to -0.003). The prevalence of low muscle density was dependent on the cutoff point and increased with age. A cutoff point of 32 HU for women and 37 HU for men and an M/S of 0.65 for women and 0.75 for men were used to define low muscle density. Low muscle density defined by those cutoff points was associated with the risk of VCF [muscle attenuation: adjusted hazard ratio (aHR) = 0.422 (95% CI: 0.256–0.696) for women; aHR = 0.391 (95% CI: 0.173–0.883) for men; M/S: aHR = 0.40 (95% CI: 0.23–0.68) for women; aHR = 0.23 (95% CI: 0.09–0.58) for men].

**Conclusion:**

Muscle density decreases with age. The muscle attenuation of 32 HU for women and 37 HU for men, an M/S of 0.65 for women and 0.75 for men, may be used to define low muscle density.

**Supplementary Information:**

The online version contains supplementary material available at 10.1007/s40520-025-02933-9.

## Introduction

Sarcopenia is an age-related disease that involves the loss of muscle mass and function. The concept of sarcopenia was first proposed by Irwin Rosenberg in 1989 [1]. The European Working Group on Sarcopenia in Older People (EWGSOP) first defined sarcopenia as a decrease in muscle mass with a decrease in muscle strength or muscle function [2]. Sarcopenia was officially encoded as a muscle disorder by the International Classification of Diseases (ICD-10) in 2016 [3]. Sarcopenia has attracted great attention in the past decade. Recently, the EWGSOP Asian Working Group on Sarcopenia (AWGS) was updated and refined the diagnosis of sarcopenia into presarcopenia, sarcopenia, and severe sarcopenia in 2019 [4, 5].

Sarcopenia is a global public health issue caused by population aging. The global prevalence of sarcopenia is 10–27% in older adults [6]. Sarcopenia may increase the risk of adverse outcomes such as falls, fractures, disability, and death [5]. Therefore, early detection of sarcopenia in these individuals is critical for the prevention of sarcopenia-related adverse outcomes.

Both the EWGSOP [2] and the AWGS [7], which were updated as EWGSOP2 [5], are recommended for diagnosing sarcopenia on the basis of measurements of a combination of muscle mass, muscle strength (grip strength), and physical performance (gait speed or timed up and go test). A recent report indicated that muscle quality might be a more relevant concept to health than muscle mass is [8]. However, how to define muscle quality has not been well studied. Intermuscular or intramuscular adiposity may be a marker of muscle quality [8]. Some studies have shown that computed tomography (CT)-based muscle density or muscle attenuation, which can reflect the degree of intermuscular or intramuscular adiposity, is associated with adverse outcomes, such as the risk of bone fracture and morality [9–12]. We speculated that CT-based muscle density or muscle attenuation may be used for sarcopenia or low-muscle-quality assays in the general population. However, normative data for muscle density across the life course in the general population have not been well studied. Moreover, there are no standard criteria for muscle density, and whether cutoff points can be derived for low muscle density evaluation is also unknown. A recent systematic review also indicated that studies should be performed with CT-derived diagnostic thresholds for myosteatosis, not only in cancer studies but also in other cohorts, including older adults [13].

CT lung cancer screening is usually recommended for people aged 50 years or older who are also susceptible to sarcopenia. It would be feasible to opportunistically detect sarcopenia during those scans because such detection does not increase additional radiation exposure or cost. In the present study, we aimed to establish a cutoff point for low muscle density evaluation. Finally, to determine the clinical relevance of the obtained cutoff point, we further explored the association between cutoff point-based low muscle density and spine compression fracture in an independent population.

## Methods

### Study population

**Fig. 1:**
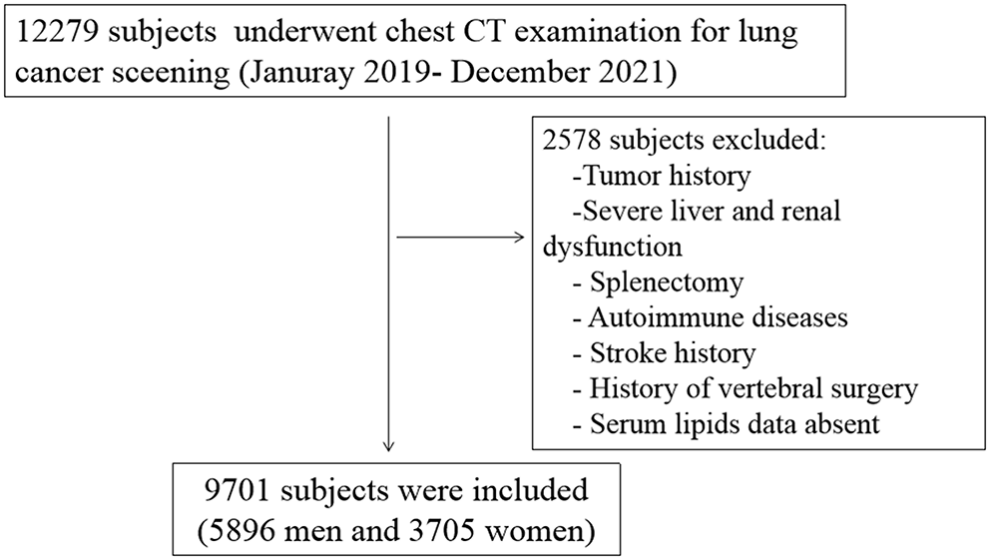
The flowchart of the study population

This retrospective multicenter study included subjects aged 50 years or older who underwent CT lung cancer screening between July 2019 and January 2021. Participants with data on liver function, kidney function, serum lipid and blood glucose levels or a history of diabetes were included. Participants with a history of tumors, severe liver dysfunction (twofold elevation in functional biomarkers), severe kidney dysfunction, autoimmune diseases (such as rheumatoid arthritis), previous stroke, or a history of vertebral surgery were excluded. The flowchart of the subject identification and selection process is shown in Fig. 1. This study was approved by the Ethics Committee of the Affiliated Hospital of Nanjing University of Chinese Medicine, and written informed consent was waived because of the retrospective design.

Were the cutoff points obtained in our study associated with longer-term clinical outcomes? We further investigated the association between low muscle density and VCF in the Chinese population in a longitudinal study. A population aged 50 years or older who did not have vertebral compression fracture (VCF) and underwent CT lung cancer screening from 2016 to 2020 at our institution was included after excluding those with a medical history of tumors, severe kidney and liver dysfunction, chronic lung diseases and pathological fracture or long-term use of steroids. This population was followed up until January 2023. Only those with at least two CT scans were included for further analysis. This population was included for assessing the association between low muscle density and VCF. VCFs were evaluated on the basis of the Genant score on sagittal images during the follow-up [14].

### Assessment of muscle attenuation

**Fig. 2:**
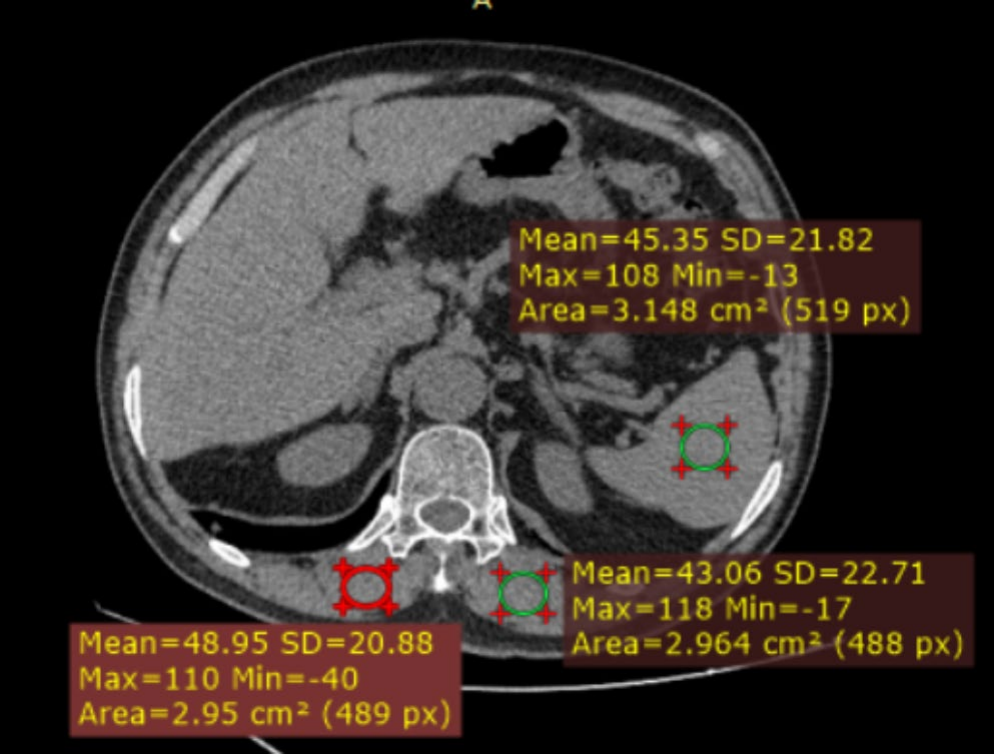
Measurement of muscle CT attenuation. **A**: Axial computed tomography image at the T11 level. **B**: Muscular and spleen CT attenuation was measured with a region of interest of 2.0 cm^2^

All CT scans were performed by two types of machines (Philips Brilliance 64; GE Optima CT680). The scan parameters were as follows: voltage, 120 kV; section thickness, 3.0 mm; matrix, 512 × 512; and reconstruction thickness, 2.0 mm. Single axial CT images were obtained from the picture archiving and communication system (PACS), and the measurement level was set at the middle level of the T11 vertebra. Muscle attenuation of the bilateral erector spinae was measured via PACS. The mean muscle attenuation (Hounsfield units [HUs]) was calculated for further analysis. An illustration of the muscle attenuation measurement is shown in Fig. 2. Spleen CT attenuation was also measured, and the muscle‒spleen ratio (MS) was calculated. Two approaches were used to define low muscle density: the T score and cutoff point. The T score, which is analogous to that used to define osteoporosis, was calculated via the following equation: (Xm-Xµ)/standard deviation (SD), where Xm is the measured muscle attenuation or MS, Xµ is the average muscle attenuation or MS of the same sex and young adults (30–40 years old), and the standard deviation (SD) is Xµ. If the T score was ≤ -2.5, low muscle density was considered. In addition, cutoff points of 32 HU, 35 HU, 37 HU, 40 HU, 42 HU and 44 HU for women and 37 HU, 40 HU, 43 HU, 46 HU, and 49 HU for men were used to define low muscle density. The cutoff points for muscle CT attenuation were selected on the basis of the 5th to 60th percentiles. The cutoff points of M/S 0.7, 0.75, 0.8, and 0.85 for men and 0.65, 0.7, 0.75, and 0.8 for women were used to define low muscle density. The cutoff points of the M/S ratio were selected on the basis of the 5th to 30th percentiles.

### Statistical analysis

The data were analyzed via SPSS 22.0 (SPSS Inc., Chicago, IL, USA). Qualitative data are presented as numbers (percentages), and quantitative data are presented as the means ± SDs. A Bland‒Altman plot was used to show the reproducibility of the measurements of muscle attenuation. Pearson correlation analysis and linear regression analysis were used to determine the associations between muscle attenuation and age. On the basis of the T score-related low muscle density, we also calculated the cutoff point for erector spinae attenuation via a receiver operating characteristic (ROC) curve. The ROC curve was also used to define the cutoff point of MS for low muscle density. All the subjects were divided into seven age groups: 20–30, 30–40, 40–50, 50–60, 60–70, 70–80 and > 80 years. A Cox proportional hazards model and hazard ratio curve were applied to determine the associations between muscle attenuation and VCF. *P* < 0.05 was considered statistically significant.

## Results

### Participant characteristics

The characteristics of the participants are shown in Table 1. A total of 9701 subjects (5896 men) were included in this study. Significant differences in age, serum lipid levels, creatinine levels, blood glucose levels, and serum albumin and total protein levels were detected between men and women (*p* < 0.01). In addition, muscle CT attenuation and the M/S ratio were significantly greater in men than in women (*p* < 0.01). The measurement reproducibility of muscle CT attenuation was good (supplemental Fig. 1), and the distributions of muscle attenuation in men and women were normal (supplemental Fig. 2).

**Table 1:** Characteristics of the participants

	Overall (*n* = 9701)	Men (*n* = 5896)	Women (*n* = 3805)	*p*
Age (year)	51.79 ± 15.46	52.54 ± 15.62	50.28 ± 14.99	< 0.01
Age groups				< 0.01
20–30	800	445	355	
30–40	2012	1182	830	
40–50	1788	1070	718	
50–60	2289	1343	946	
60–70	1528	973	555	
70–80	960	656	304	
80	324	229	95	
Serum Albumin (g/L)	41.04 ± 3.20	41.13 ± 3.18	40.91 ± 3.23	< 0.01
Total protein (g/L)	67.91 ± 4.56	67.72 ± 4.62	68.21 ± 4.45	< 0.01
AST (U/L)	24.33 ± 23.38	25.17 ± 15.10	23.01 ± 32.25	< 0.01
TG (µmol/L)	1.56 ± 1.32	1.73 ± 1.49	1.28 ± 0.94	< 0.01
TC (µmol/L)	4.67 ± 0.97	4.59 ± 0.95	4.79 ± 0.98	< 0.01
HDL (µmol/L)	1.51 ± 0.35	1.41 ± 0.31	1.67 ± 0.35	< 0.01
LDL (µmol/L)	2.95 ± 0.81	2.97 ± 0.81	2.91 ± 0.82	< 0.01
Creatinine (mmol/L)	68.78 ± 20.18	73.00 ± 23.35	62.21 ± 11.03	< 0.01
Blood Glucose (µmol/L)	4.66 ± 1.04	4.75 ± 1.11	4.53 ± 0.90	< 0.01
Diabetes	209	158	51	< 0.01
Muscle CT attenuation (HU)	44.79 ± 7.17	45.90 ± 7.23	43.29 ± 6.87	< 0.01
M/S	0.91 ± 0.15	0.92 ± 0.15	0.90 ± 0.15	< 0.01

AST: aspartate aminotransaminase; HDL-c: high-density lipoprotein cholesterol; HU = Hounsfield units; LDL-c: low-density lipoprotein cholesterol; M/S: muscle/spleen ratio; TC: total cholesterol; TG: triacylglycerol

The baseline characteristics of the participants included in the VCF assessment are shown in supplemental Table 1. A total of 2006 subjects were followed up for 2–6 years. VCF was observed in 98 participants. VCFs tend to occur in women and aged individuals. The CT values of the erector spinae in participants with bone fractures were lower than those in participants without fractures (*p* < 0.001). The prevalence of low muscle CT attenuation in participants with VCF was greater than that in those without VCF (*p* < 0.05 in men; *p* < 0.01 in women).

### Associations between age and muscle attenuation and the M/S ratio

**Fig. 3:**
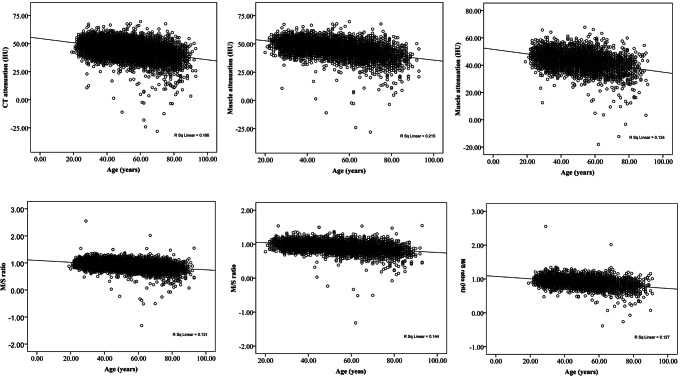
The correlations between age and muscle attenuation (upper) and the muscle/spleen ratio (low) in the total population, men and women

Muscle attenuation was negatively correlated with age in the total population (*r* = -0.40, *p* < 0.01), men (*r* = -0.46, *p* < 0.01) and women (*r* = -0.37, *p* < 0.01) (Fig. 3). Muscle attenuation decreased with increasing age (Supplemental Fig. 3). Multiple linear regression analysis also revealed that muscle attenuation was associated with age in the total population (β = -0.19, 95% CI: -0.21 to -0.18), men (β = -0.20, 95% CI: -0.21 to -0.19) and women (β = -0.17, 95% CI: -0.19 to -0.16) (Table 2).

**Table 2:** Linear regression analysis showing the associations between muscle CT attenuation and clinical variables

	Total population		Men		Women	
	β (95%CI)	*p*	β (95%CI)	*p*	β (95%CI)	*p*
Age (year)	-0.19 (-0.21 to -0.18)	< 0.001	-0.20 (-0.21 to -0.19)	< 0.001	-0.17 (-0.19 to -0.16)	< 0.001
Serum Albumin (g/L)	0.059 (0.013 to 0.11)	0.013	0.078 (0.017 to 0.14)	0.012	0.035 (-0.04 to 0.11)	0.35
Total protein (g/L)	-0.026 (-0.06 to 0.006)	0.11	-0.015 (-0.06 to 0.03)	0.48	-0.04 (-0.10 to 0.008)	0.10
AST (U/L)	-0.002 (-0.008 to 0.003)	0.42	-0.002 (-0.013 to 0.008)	0.66	-0.002 (-0.009 to 0.004)	0.45
Serum Creatinine (µmol/L)	0.007 (0.00 to 0.014)	0.036	0.003 (-0.004 to 0.01)	0.036	0.025 (0.006 to 0.04)	0.01
Blood Glucose (µmol/L)	-0.075 (-0.21 to 0.06)	0.26	-0.035 (-0.19 to 0.12)	0.66	-0.16 (-0.41 to 0.08)	0.19
TG (µmol/L)	0.24 (0.12 to 0.36)	< 0.01	0.17 (0.04 to 0.31)	0.014	0.25 (-0.02 to 0.52)	0.54
TC (µmol/L)	-0.185 (-0.48 to 0.11)	0.21	0.13 (-0.25 to 0.51)	0.21	-0.63 (-1.09 to -0.17)	0.007
HDL (µmol/L)	-0.70 (-1.20 to -0.20)	< 0.01	-1.03 (-1.70 to -0.37)	< 0.01	-0.25 (-1.00 to 0.53)	0.54
LDL (µmol/L)	0.81 (0.50 to 1.18)	< 0.01	0.49 (0.10 to 0.88)	0.015	1.22 (0.73 to 1.72)	< 0.01
Gender (men vs. women)	2.60 (2.30 to 2.90)	< 0.001				

AST: aspartate aminotransaminase; CI: confidence interval; HDL-c: high-density lipoprotein cholesterol; LDL-c: low-density lipoprotein cholesterol; TC: total cholesterol; TG: triacylglycerol

M/S was also negatively correlated with age in the total population (*r* = -0.36, *p* < 0.01), men (*r* = -0.38, *p* < 0.01) and women (*r* = -0.35, *p* < 0.01) (Fig. 1). The M/S ratio decreased with increasing age (Supplemental Fig. 3). Multiple linear regression analysis demonstrated that M/S was associated with age in the total population (β = -0.004, 95% CI: -0.004 to -0.003), men (β = -0.003, 95% CI: -0.004 to -0.003) and women (β = -0.004, 95% CI: -0.004 to -0.003) (supplemental Table 2).

### Prevalence of low muscle attenuation

**Fig. 4:**
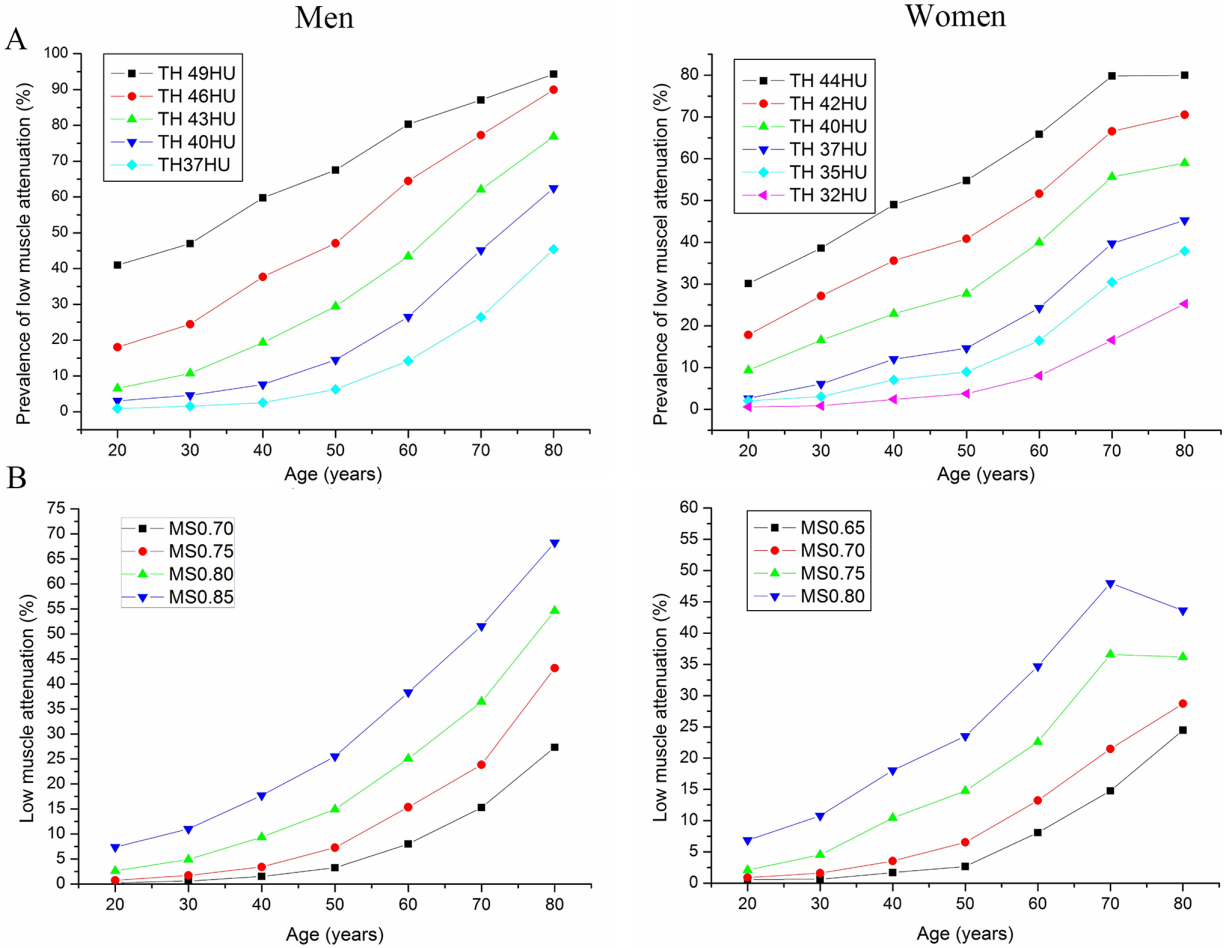
The prevalence of low muscle attenuation in men and women based on muscle attenuation (**A**) and the M/S ratio (**B**) in different age groups. The cutoff points for low muscle density were set as 37, 40, 43, 46, 49 HU or 0.75, 0.8, 0.85, 0.9 of M/S for men and 32, 35, 37, 40, 42, 44 HU or 0.65, 0.7, 0.75, 0.8 of M/S for women, respectively. The cutoff points for muscle CT attenuation were selected on the basis of the 5th to 60th percentiles. The cutoff points of the M/S ratio were selected on the basis of the 5th to 30th percentiles. The prevalence of sarcopenia was strongly associated with the cutoff points

Next, we investigated the prevalence of low muscle attenuation in different age groups (Fig. 4). The cutoff points for low muscle density were 37, 40, 43, 46, and 49 HU for men and 32, 35, 37, 40, 42, and 44 HU for women (Fig. 4A). The prevalence of low muscle attenuation increased with age. The prevalence was also dependent on the cutoff point. A high cutoff point was associated with a higher prevalence. The prevalence was the lowest when the cutoff point was 37 HU for men and 32 HU for men. The prevalence of low muscle density was 0.95%, 1.54%, 2.55%, 6.27%, 14.37%, 26.67% and 45.61% for men and 0.6%, 0.86%, 2.39%, 3.85%, 8.24%, 16.7% and 25.2% for women in different age groups (20–30, 30–40, 40–50, 50–60, 60–70, 70–80, and > 80 years, respectively).

The cutoff points for low muscle density were set as 0.7, 0.75, 0.8, and 0.85 for men and 0.65, 0.7, 0.75, and 0.8 for women. The prevalence of M/S ratio-based low muscle attenuation also increased with age (Fig. 4B). The prevalence of sarcopenia was strongly associated with the cutoff points. The prevalence was the lowest when the cutoff point was 0.75 for men and 0.65 for men, which was relatively close to the data reported at present.

**Fig. 5:**
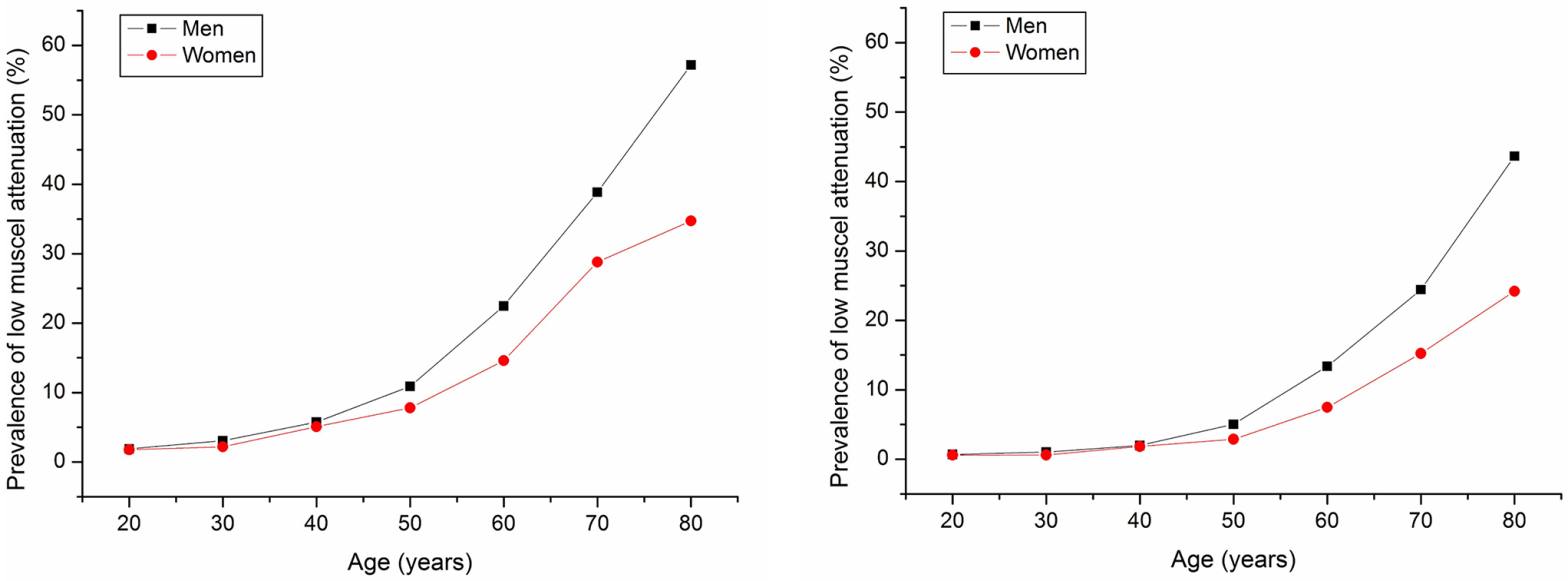
The prevalence of low muscle attenuation in men and women according to the T score method. If the T score was ≤ -2.5, low muscle density was considered. **A**: Based on muscle attenuation; **B**: based on the muscle/spleen ratio

Figure 5 further shows the T score (< -2.5) based on low muscle density. Similarly, the incidence of low muscle attenuation increased with age. The prevalence rates of low muscle density were 0.7%, 1.03%, 1.98%, 5.06%, 13.48%, 24.61% and 43.86% for men and 0.6%, 0.60%, 1.83%, 3.00%, 7.68%, 15.28% and 24.21% for women in different age groups (20–30, 30–40, 40–50, 50–60, 60–70, 70–80, and > 80 years, respectively) on the basis of T scores of muscle attenuation < -2.5. The prevalence was higher in males than in females for subjects aged 50 years or older. Interestingly, the T score of attenuation-based prevalence was similar to the cutoff point of 32 HU for women and 37 HU for men. The kappa test also revealed good agreement between the two prevalence rates (Kappa = 0.98 for men, 0.96 for women) (supplemental Fig. 5). Similarly, the T score of the M/S-based prevalence was close to the cutoff point of 0.65 for women and 0.75 for men (kappa = 1.0 for men and 0.95 for women) (supplemental Fig. 6).

### ROC curve

We subsequently determined the cutoff points for muscle attenuation and the muscle/spleen ratio via receiver operating characteristic (ROC) curve analysis on the basis of the T score-defined low muscle attenuation. A cutoff point of 31.6 HU for women and 36.5 HU for men was obtained to define low muscle density, with areas under the curve (AUCs) of 1.00 (specificity of 100% and sensitivity of 100%) and 1.00 (specificity of 94% and sensitivity of 91%) (supplemental Fig. 7), respectively. In addition, cutoff points of 0.75 for men and 0.645 for women were obtained for M/S to define low muscle density (AUC = 1.00, specificity 100% and sensitivity 100% in men; AUC = 1.00, sensitivity 1.00%, specificity 1.00% (supplemental Fig. 8)).

### The association between low muscle quality and the risk of fracture

The hazard ratio curve revealed that the risk of VCF in subjects with low muscle attenuation was significantly greater than that in those with high muscle attenuation (*P* < 0.01, Supplemental Fig. 9). High CT attenuation (> 37 HU for men and > 32 HU for women) was a significant protective factor in the age- and BMD-adjusted model, with hazard ratios (HRs) of 0.422 (95% CI: 0.256–0.696, *P* < 0.01) for women and 0.391 (95% CI: 0.173–0.883, *P* = 0.024) for men (Table 3). A low M/S ratio was also significantly associated with the risk of VCF in the age- and osteoporosis-adjusted model (aHR = 0.40 (95% CI: 0.23–0.68) for women, aHR = 0.23 (95% CI: 0.09–0.58) (Table 3).

**Table 3:** Relationships between muscle attenuation (categorical data) or the muscle/spleen ratio and compression fracture in women and men

			Univariable	*P*	Multivariables	*P*
Muscle Attenuation	Women	CT value of vertebrae	0.962 (0.955–0.969)	< 0.001	0.974 (0.965–0.983)	< 0.001
		CT value of Muscle ≥ 32 HU	0.149 (0.092–0.239)	< 0.001	0.422 (0.256–0.696)	0.001
	Men	CT value of vertebrae	0.957 (0.946–0.969)	< 0.001	0.962 (0.950–0.975)	< 0.001
		CT value of Muscle ≥ 37 HU	0.168 (0.076–0.367)	< 0.001	0.391 (0.173–0.883)	0.024
Muscle/spleen ratio						
	Women	MS < 0.65	6.06 (3.59–10.22)	< 0.001	2.50(1.46–4.29)	0.001
		Osteoporosis	25.26 (9.22–69.25)	< 0.001	9.38 (3.30–26.60)	< 0.001
	Men	MS < 0.75	8.48 (3.66–19.65)	< 0.001	4.31(1.73–10.76)	0.002
		Osteoporosis	9.83 (4.34–22.29)	< 0.001	4.76 (1.88–11.96)	< 0.001

Note.—Data in parentheses are 95% CIs. CT = computed tomography; HU = Hounsfield units

A further adjustment for age and muscle area was performed in multivariate Cox regression analysis

## Discussion

The EWGSOP2 indicates that muscle strength and muscle quantity or quality are the two determinants for sarcopenia diagnosis. Muscle quantity can be assessed by DXA, BIA, CT or MRI. However, how to define muscle quality is still unclear. Intermuscular or intramuscular adiposity may be a marker of muscle quality [8]. Muscle CT attenuation could reflect the degree of intermuscular or intramuscular adiposity. Therefore, muscle CT attenuation may be used as a marker for muscle quality. In this study, on the basis of a large population that underwent CT lung cancer screening, we found that muscle CT attenuation decreased with age. On the basis of T score-related low muscle density, we obtained cutoff points of 32 HU for women and 37 HU for men and M/S values of 0.65 for women and 0.75 for men to define low muscle density. We also showed that those cutoff points were associated with clinical outcomes from the aspect of VCF.

The associations between muscle attenuation and clinical outcomes, such as fracture [12, 15] and mortality [16–19], have been widely studied. Recently, a study used muscle density as a measure of muscle quality in patients who underwent surgery [19]. However, whether muscle CT attenuation can be used for sarcopenia or low-muscle-quality assays in the general population is still unknown. Sarcopenia is an age-related disease. If a marker can be used for sarcopenia or low-muscle-quality assays, it should decrease with age. Interestingly, our data revealed that muscle CT attenuation and M/S decreased with age in both men and women. These trends indicate that muscle CT attenuation may have great potential in the evaluation of sarcopenia.

The T score has been used to define osteoporosis. This method may also be useful for defining low muscle quality or muscle attenuation. We calculated the T score of muscle attenuation and M/S and then defined low muscle attenuation as a T score < -2.5. The prevalence of low muscle attenuation was 0.6-24.1% in women and 0.7-43.8% in men on the basis of a T score of muscle attenuation <-2.5. Similar trends were observed when the T score of M/S was <-2.5. There is a large difference in the prevalence of sarcopenia across studies because different definitions, diagnostic criteria, and measurement techniques are used [18]. Our data are consistent with findings that the prevalence of sarcopenia is greater in men than in women. The T score-based low muscle density data are also similar to previous data concerning sarcopenia in the Chinese population (5.1–21.0% in men and 4.1–16.3% in women) [20]. CT attenuation or the M/S-based T score may be applicable markers for defining low muscle quality.

Muscle CT attenuation has been used for muscle quality assessment [21]. We subsequently speculated that cutoff points exist for muscle attenuation or M/S to define low muscle quality. Therefore, we calculated the cutoff point via an ROC curve considering T score-based low muscle quality as the gold standard. We obtained reference levels of 37 HU and 0.75 M/S for men and 32 HU and 0.65 M/S for women in defining low muscle density. In addition, we also calculated the prevalence of low muscle density on the basis of differences in muscle attenuation. Interestingly, we observed that the prevalence of low muscle density based on 37 HU or MS/S of 0.75 for men and 32 HU or M/S of 0.65 for women was close to that of the T score-based prevalence. The kappa test also revealed good agreement in terms of prevalence between the cutoff point and T score approaches. Those analyses revealed that 37 HU or MS/S of 0.75 for men and 32 HU or M/S of 0.65 for women were acceptable normal reference levels for low muscle density. To our knowledge, no study has reported the cutoff point of muscle attenuation for sarcopenia or muscle quality assays in the general population. The cutoff points obtained in our study should be validated in other populations.

AWGS-defined sarcopenia is associated with clinical outcomes, such as physical limitations at 4 years and 10-year mortality [4]. Were the cutoff points obtained in our study associated with longer-term clinical outcomes? We further investigated the association between low muscle density and VCF in the Chinese population. Interestingly, the results indicated that low muscle density, defined by our cutoff points, was significantly associated with the risk of CVF. These results showed that the reference levels obtained in our study have clinical value.

Recently, incidental findings from CT lung cancer screening have attracted increased attention [22–25]. However, sarcoepnia was not considered in those studies. One important reason is the lack of measurement thresholds for low muscle mass or muscle density. Our study provided cutoff points for muscle density. On the basis of these cutoff points, sarcopenia or low muscle density may also be evaluated during CT-based lung cancer screening, especially for older individuals. The HGS and SARC-F [26] have potential roles in screening for sarcopenia. Our data demonstrated that muscle CT attenuation or the M/S ratio are also valuable for sarcopenia management.

Our study has several advantages. First, this was a large multicenter study to determine the cutoff point of muscle CT attenuation for sarcopenia or low muscle quality in the general population. Second, we used several methods to calculate the cutoff points. Interestingly, the cutoff points obtained from the different methods were almost identical. Third, we also calculated the cutoff point of the M/S ratio. Finally, our study revealed that low muscle density, defined by our cutoff points, was significantly associated with HGS and clinical outcome. Our study has several limitations. First, we only measured muscle attenuation in the erector spinae. Whether our cutoff point is applicable for other muscles should be validated. Second, two different CT devices were used for muscle evaluation. There may be measurement variations between different CT devices. However, the variations were not evaluated because we could not ask people to undergo two CT scans at the two different institutions. Most of our population (*n* > 8000) was from the same center (Affiliated Hospital of Nanjing University of Chinese Medicine). The variations between different CT devices may not influence the conclusion. Third, we did not find agreement between low muscle density and AWGS-defined sarcopenia or the SARC-F [27, 28] because they were not assessed in our population. Previous studies have shown that CT-based muscle attenuation is also related to muscle strength independent of muscle mass [29, 30]. Oba et al. [31] reported that muscle attenuation was correlated with physical performance, including performance on a standing-up test (*r* =-0.40) and walking speed (*r* = -0.43). Fourth, the study is based only on the Chinese population, and the generalizability of the obtained cutoff values for different ethnic groups or geographic regions is limited. Validation studies should be conducted in different populations. Finally, we reported only the long-term outcomes of VCFs. Whether the low muscle density defined in our study is associated with other outcomes, such as mortality and slowness, should be investigated.

## Conclusion

Our study revealed that muscle attenuation and M/S decreased with age. The prevalence of low muscle density increases with age. A T score < -2.5 for muscle attenuation or M/S may be useful for defining low muscle density. We also obtained cutoff points for muscle attenuation (37 HU for men and 32 HU for women) and M/S (0.75 for men and 0.65 for women) for low muscle density evaluation. These cutoff points may be used for low muscle density assessment during chest CT scans.

## Electronic supplementary material

Below is the link to the electronic supplementary material.

Supplementary Material 1

## Data Availability

The datasets used and/or analyzed during the current study available from the corresponding author on reasonable request.
