# Supplementary material for: Opportunistic muscle density assay during CT lung cancer screening for low muscle quality evaluation in older adults: a multicenter study
Source: Aging Clin Exp Res. 2025 Feb 22;37(1):41. doi: 10.1007/s40520-025-02933-9 (PMC11846760; doi:10.1007/s40520-025-02933-9)

**Opportunistic muscle density assay during CT lung cancer screening for low muscle quality evaluation in older adults: A multicenter study**

**Supplemental Table 1 Characteristic of participants in fracture study**

|  | Total  (n = 2006) | None-fractures  (n = 1908) | Fractures  (n = 98) | p |
| --- | --- | --- | --- | --- |
| Age (years) | 62.40 ± 9.56 | 61.87 ± 9.30 | 72.87 ± 8.52 | < 0.001 |
| Sex (women) | 844 | 771 | 73 | < 0.001 |
| CT value of vertebral body (HU) | 145.39 ± 45.24 | 148.46 ± 43.7 | 85.71 ± 29.09 | < 0.001 |
| Low bone mass (< 143 HU) | 987 | 893 | 94 | < 0.001 |
| CT value of muscle (HU) | 41.94 ± 8.69 | 42.48 ± 8.39 | 31.47 ± 7.79 | < 0.001 |
| Low CT value |  |  |  | < 0.05 |
| Men(< 37 HU) | 187 | 174 | 13 |  |
| Women(< 32 HU) | 182 | 136 | 46 | < 0.01 |
| M/S | 0.86 ± 0.35 | 0.87 ± 0.35 | 0.65 ± 0.16 | < 0.01 |
| Albumin (g/L) | 40.77 ± 3.12 | 40.80 ± 3.11 | 40.06 ± 3.10 | 0.018 |
| Total protein (g/L) | 67.66 ± 4.50 | 67.64 ± 4.51 | 68.16 ± 4.21 | 0.458 |
| AST (U/L) | 24.02 ± 10.72 | 23.83 ± 7.14 | 27.72 ± 36.88 | 0.748 |
| Creatinine (μmol/L) | 76.10 (65.00,88.00) | 76.35 (65.00,87.50) | 73.00 (62.88,90.98) | 0.447 |
| HDL-c (μmol/L) | 1.50 ± 0.35 | 1.50 ± 0.35 | 1.64 ± 0.35 | <0 .001 |
| Blood glucose (μmol/L) | 5.55 ± 1.39 | 5.55 ± 1.40 | 5.56 ± 1.20 | 0.299 |
| TC (μmol/L) | 4.79 ± 1.03 | 4.79 ± 1.03 | 4.71 ± 1.02 | 0.538 |
| LDL-c (μmol/L) | 2.95 ± 0.82 | 2.96 ± 0.83 | 2.82 ± 0.78 | 0.086 |
| TG (μmol/L) | 1.29 (0.91,1.86) | 1.29 (0.91,1.87) | 1.30 (0.87,1.52) | 0.073 |

AST: Aspartate aminotransaminase; HDL-c: High density lipoprotein cholesterol; LDL-c: Low density lipoprotein cholesterol; TC: Total cholesterol; TG: Triacylglycerol

Supplemental Table 2 Linear regression analysis to show the association between ratio of muscle/spleen CT attenuation and clinical variables

|  | Total population |  | Men |  | Women |  |
| --- | --- | --- | --- | --- | --- | --- |
|  | β (95%CI) | p | β (95%CI) | p | β (95%CI) | p |
| Age (year) | -0.004  (-0.004 to -0.003) | <0.001 | -0.003  (-0.004 to -0.003) | <0.001 | -0.004  (-0.004 to -0.003) | <0.001 |
| Serum Albumin (g/L) | 0.00  (-0.001 to 0.001) | 0.54 | 0.00007  (-0.001 to 0.001) | 0.90 | 0.000  (-0.002 to 0.001) | 0.54 |
| Serum Creatinine (μmol/L) | 0.000017  (0.00 to 0.00) | 0.81 | 0.00001  (0.000 to 0.000) | 0.81 | 0.000  (0.000 to 0.001) | 008 |
| Blood Glucose (μmol/L) | -0.002  (-0.005 to 0.001) | 0.22 | -0.001  (-0.005 to 0.002) | 0.47 | -0.003  (-0.008 to 0.002) | 0.29 |
| TG (μmol/L) | 0.001  (-0.002 to 0.003) | 0.61 | 0.000  (-0.003 to 0.002) | 0.76 | 0.002  (-0.003 to 0.008) | 0.42 |
| TC (μmol/L) | 0.001  (-0.005 to 0.007) | 0.21 | 0.008  (0.000 to 0.016) | 0.04 | -0.009  (--0.019 to 0.01) | 0.074 |
| HDL (μmol/L) | -0.016  (-0.027 to -0.005) | 0.004 | -0.023  (-0.037 to -0.009) | 0.001 | -0.005  (-023 to 0.012) | 0.53 |
| LDL (μmol/L) | 0.10  (0.004 to 0.016) | 0.003 | 0.004  (-0.005 to 0.002) | 0.40 | 0.021  (0.010 to 0.03) | <0.01 |
| Gender (men vs women) | 0.03 (0.024 to 0.037) | < 0.001 |  |  |  |  |

CI: confidence interval; HDL-c: High density lipoprotein cholesterol; LDL-c: Low density lipoprotein cholesterol; TC: Total cholesterol; TG: Triacylglycerol

Supplemental Figure 1. The measurement reproducibility of muscle CT attenuation between two persons.


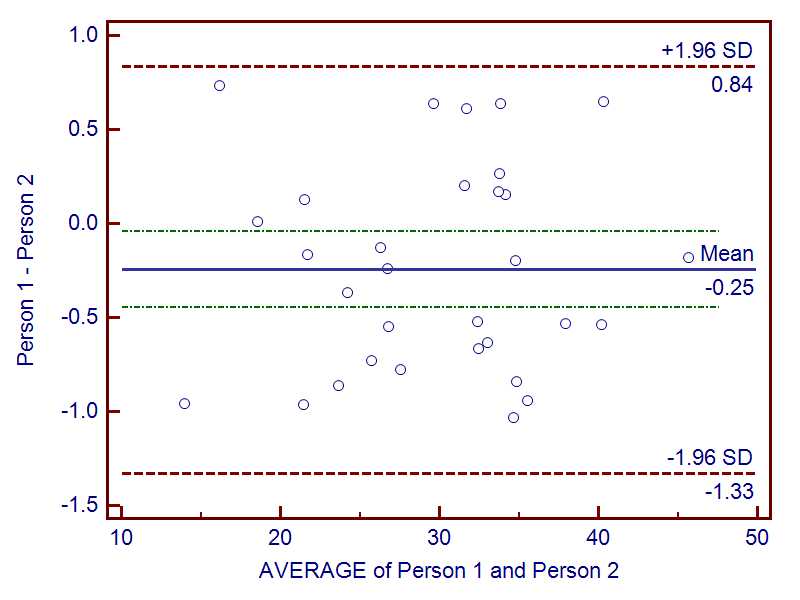


Supplemental Figure 2. The distribution of muscle attenuation in men (A) and men (B)


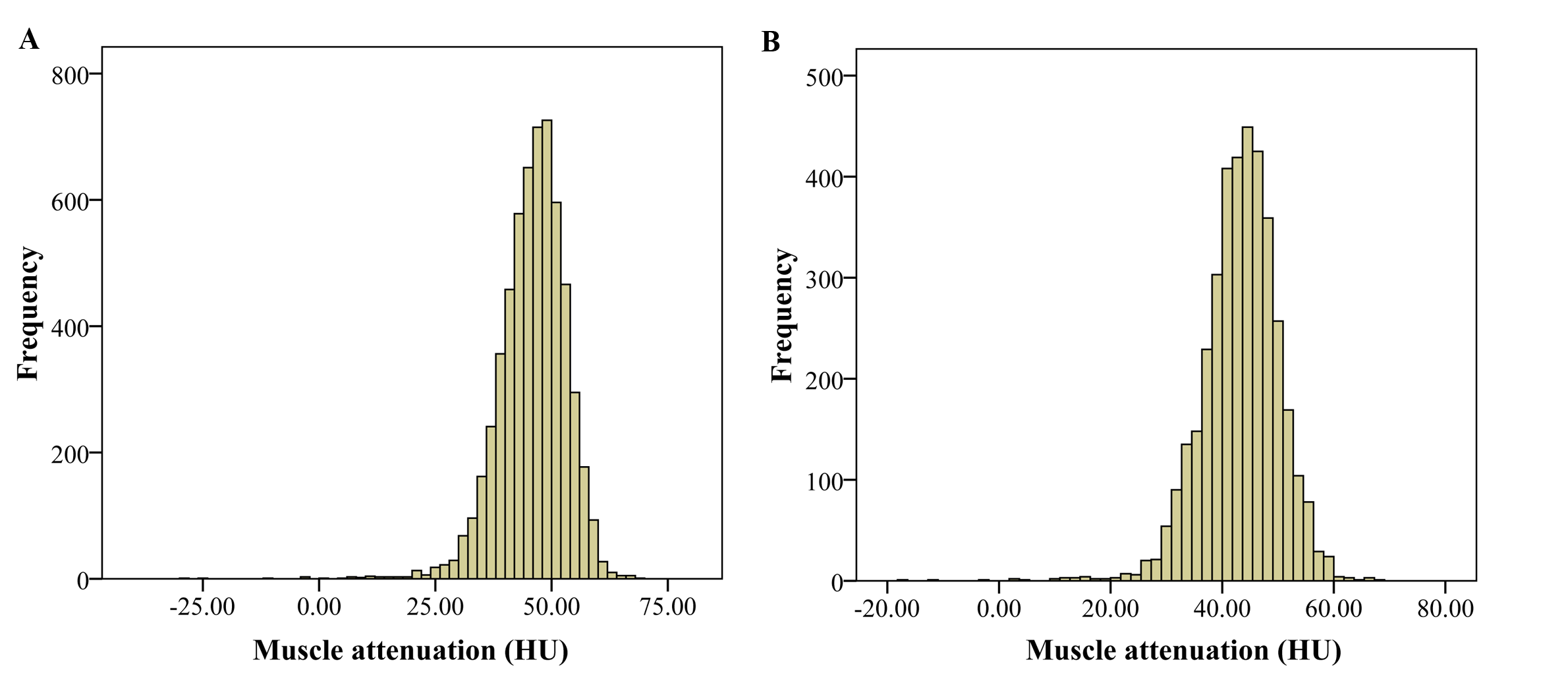


Supplemental Figure 3. The muscle attenuation (A) and muscle/spleen ratio (B) in different age groups.


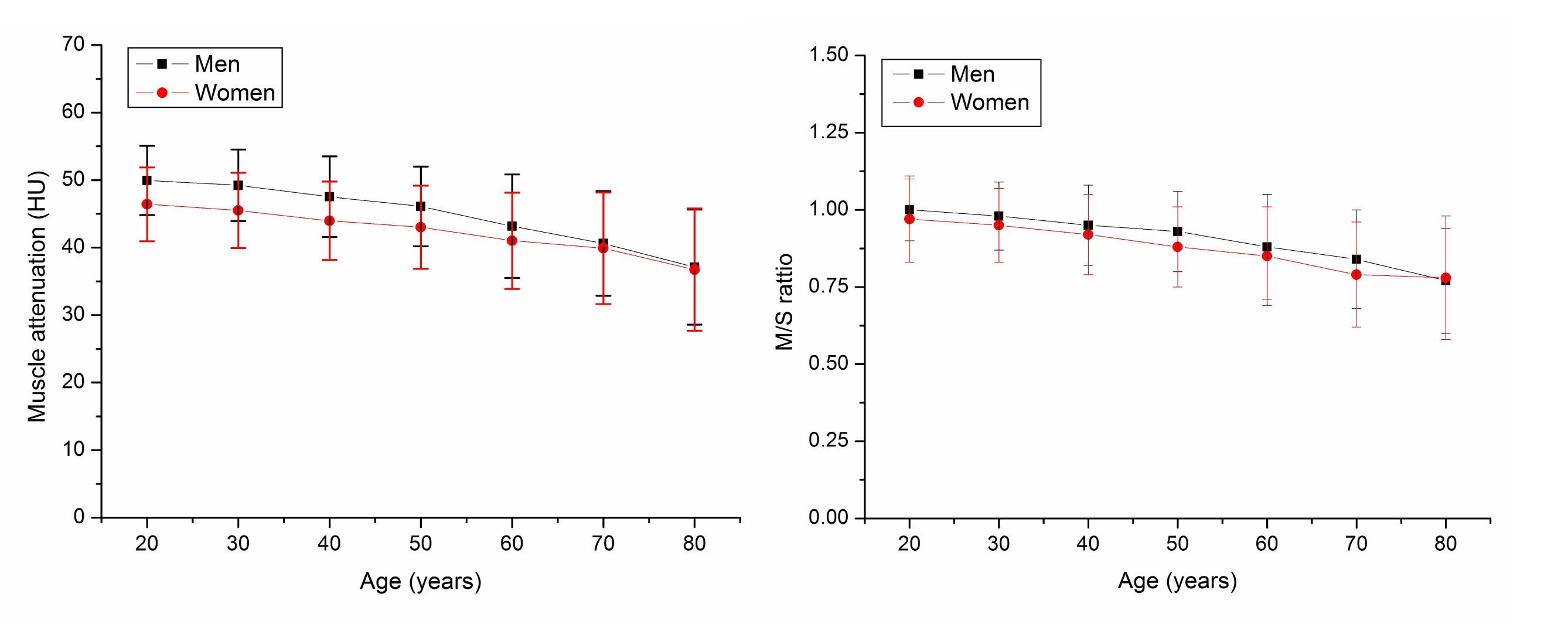


Supplemental Figure 4. The agreement between T score (<- 2.5) and cutoff point (37 HU for men and 32 HU for women) in defining low muscle attenuation in men and women.


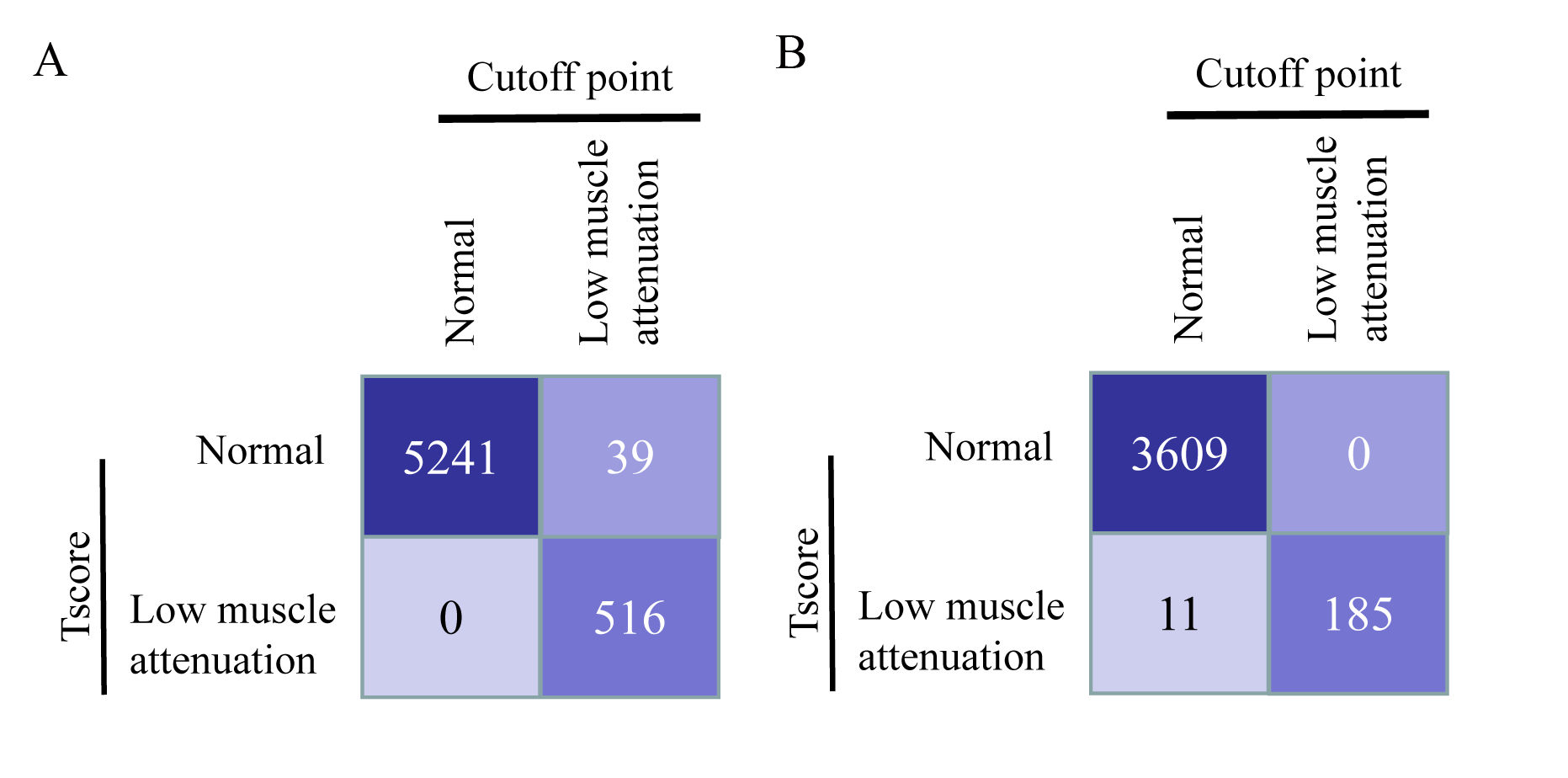


Supplemental Figure 5. The agreement between T score of M/S (<- 2.5) and cutoff point (0.75 for men and 0.65 for women) in defining low muscle attenuation in men and women.


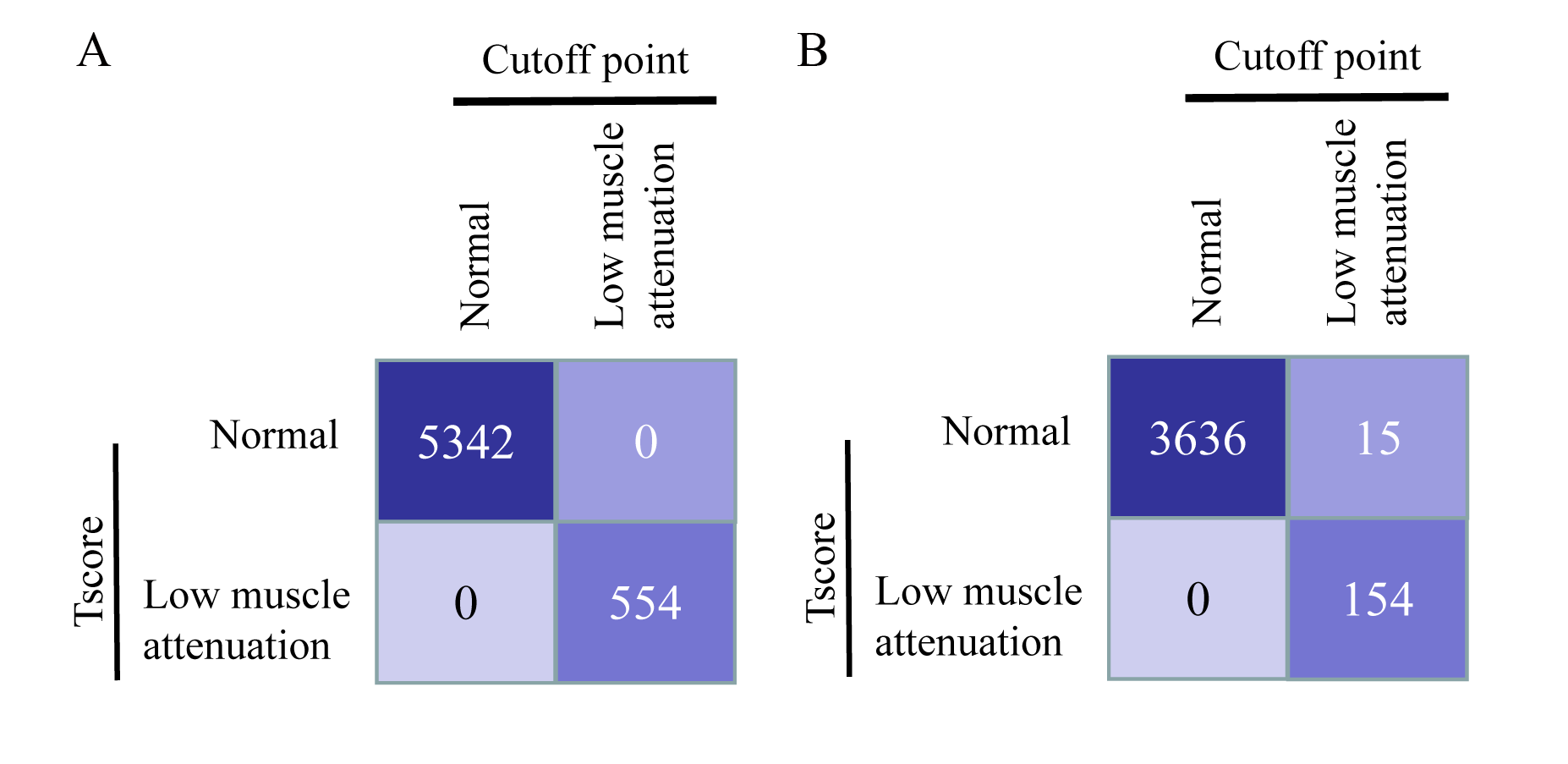


Supplemental Figure 6. ROC curve to determine cutoff point of muscle/spleen ratio based on T score defined low muscle density in men and women.


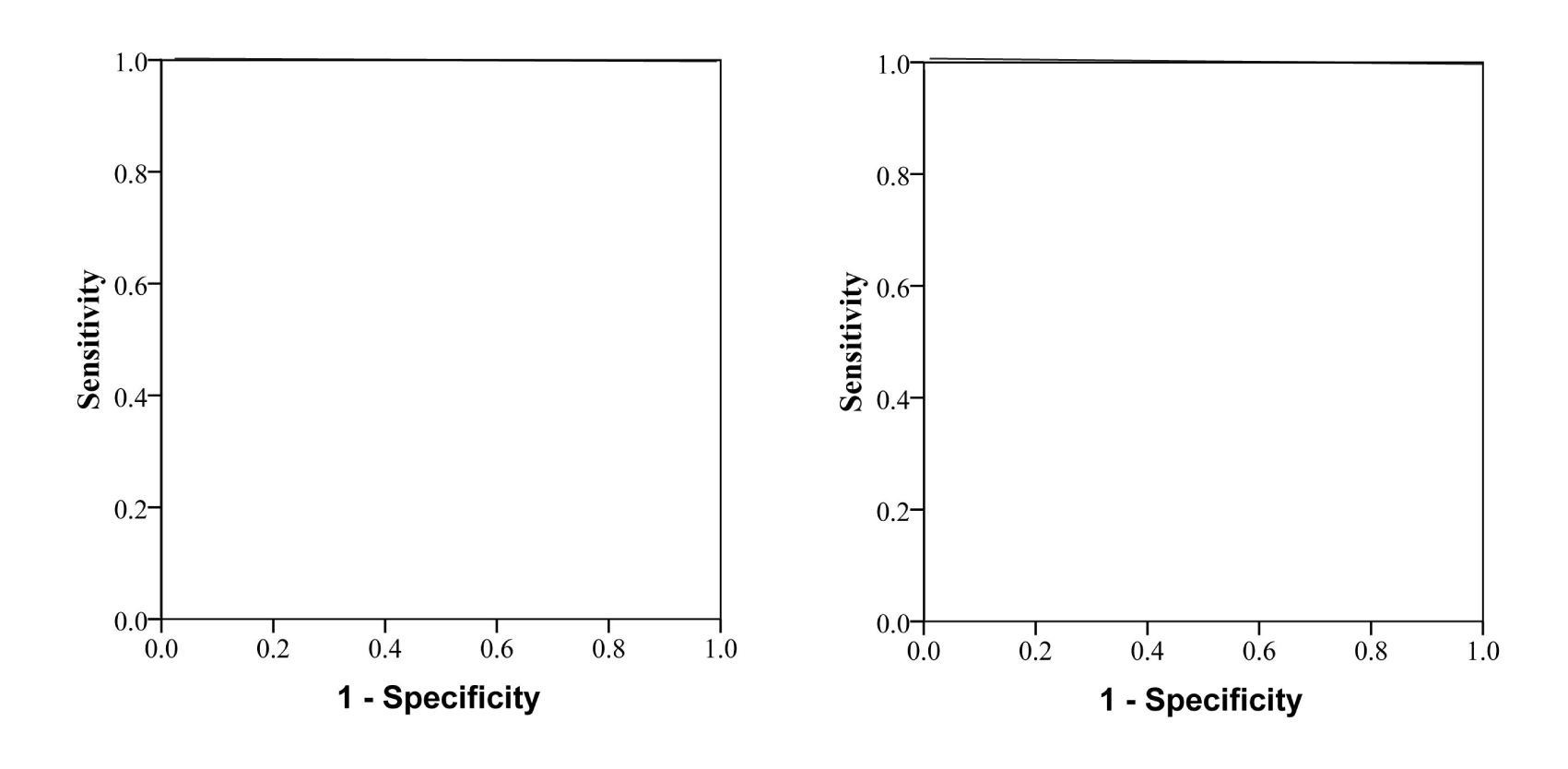


Supplemental Figure 7. ROC curve to determine cutoff point of muscle attenuation based on T score-defined low muscle density in men and women.


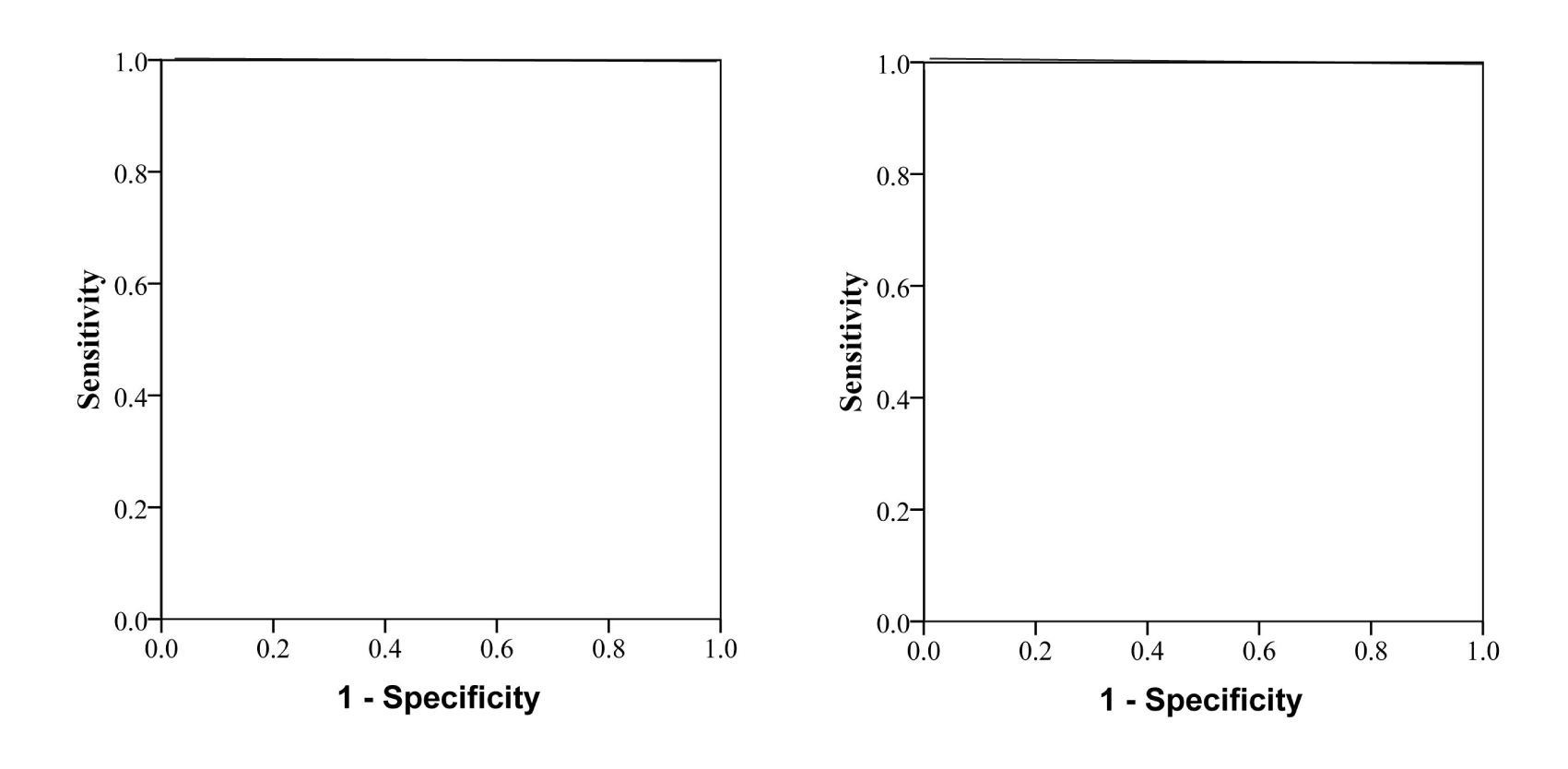


Supplemental Figure 10. ROC curve to determine cutoff point of muscle/spleen ratio based on T score-defined low muscle density in women (A) and men (B).


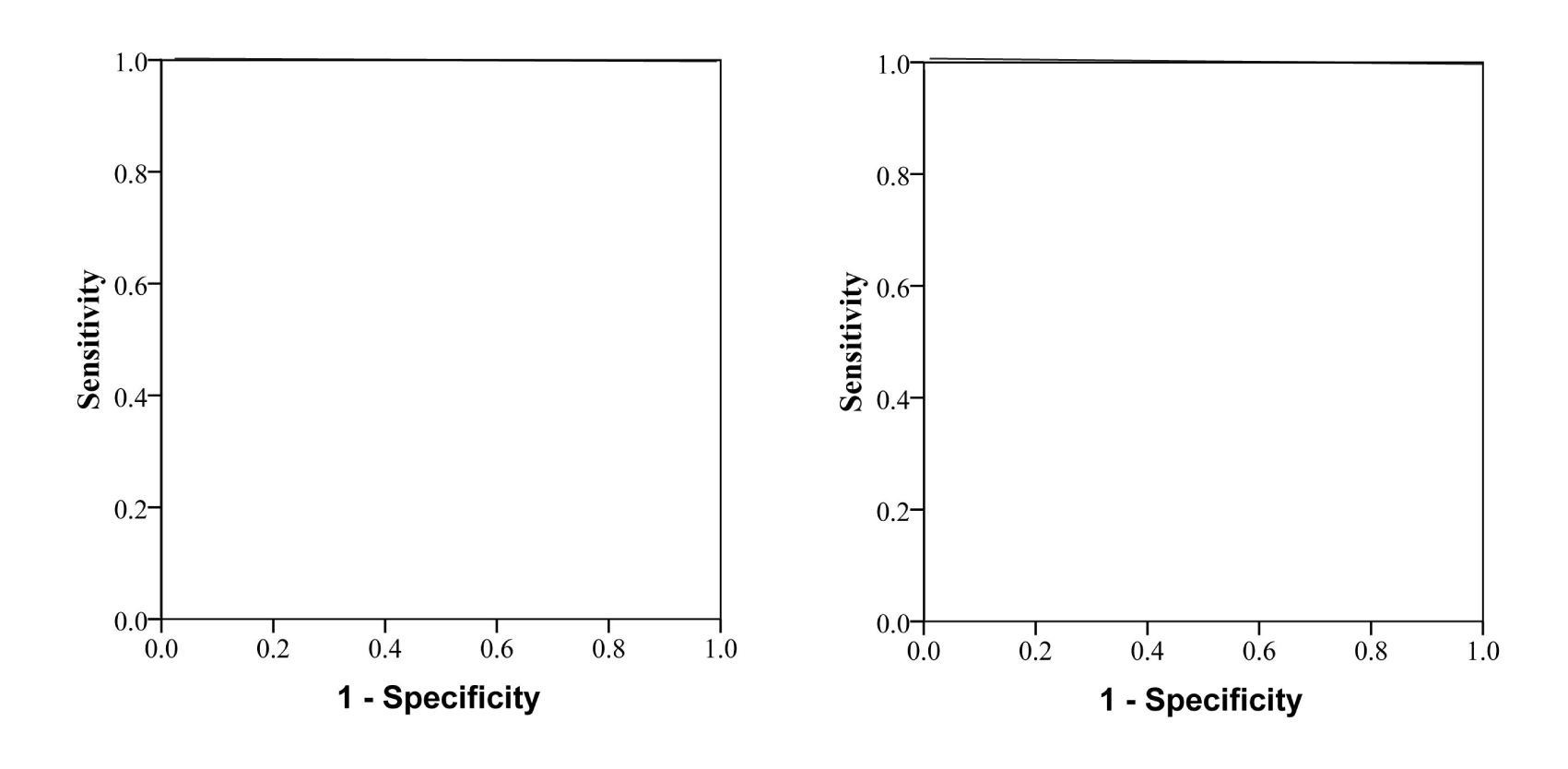


Supplemental Figure 9. The cumulative fracture risk in men (A) and women (B) with low and high muscle attenuation.


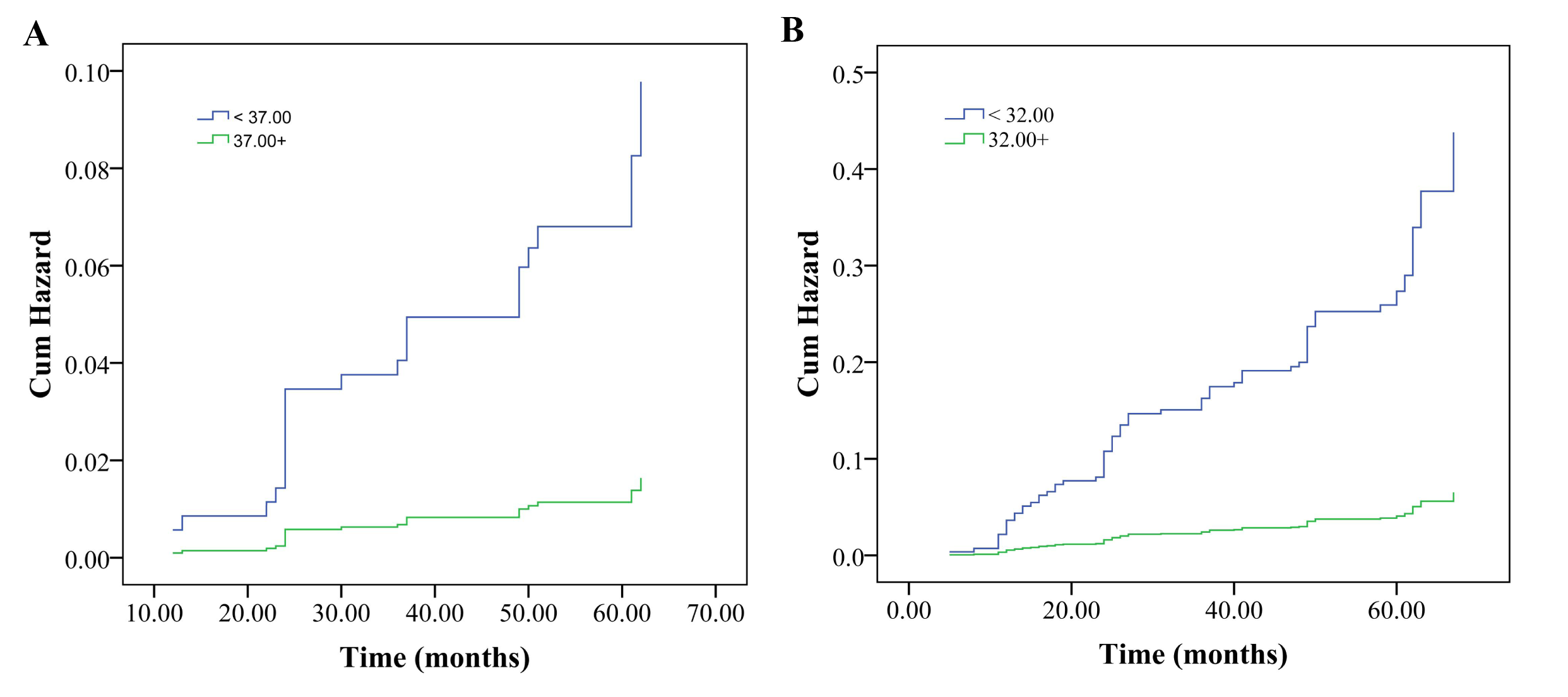

Supplement: Supplementary file 1 — Supplementary Material 1 [file 40520_2025_2933_MOESM1_ESM.docx]
